# Combinatorial regimens of chemotherapeutic agents: A new perspective on raising the heat of the tumor immune microenvironment

**DOI:** 10.3389/fphar.2022.1035954

**Published:** 2022-10-11

**Authors:** Jingyang Liu, Yang Yu, Cun Liu, Chundi Gao, Jing Zhuang, Lijuan Liu, Qibiao Wu, Wenzhe Ma, Qiming Zhang, Changgang Sun

**Affiliations:** ^1^ College of First Clinical Medicine, Shandong University of Traditional Chinese Medicine, Jinan, China; ^2^ College of Traditional Chinese Medicine, Shandong University of Traditional Chinese Medicine, Jinan, China; ^3^ College of Traditional Chinese Medicine, Weifang Medical University, Weifang, China; ^4^ Department of Oncology, Weifang Traditional Chinese Hospital, Weifang, China; ^5^ Department of Special Medicine, School of Basic Medicine, Qingdao University, Qingdao, China; ^6^ Faculty of Chinese Medicine, Macau University of Science and Technology, Macau, Macau SAR, China; ^7^ State Key Laboratory of Quality Research in Chinese Medicine, Macau University of Science and Technology, Macau, Macau SAR, China; ^8^ Department of Experimental Research Center, China Academy of Chinese Medical Sciences, Beijing, China

**Keywords:** chemotherapeutic agents, combinatorial regimens, immunogenic cell death, tumor immune microenvironment, cancer therapy

## Abstract

Harnessing the broad immunostimulatory capabilities of chemotherapy in combination with immune checkpoint inhibitors has improved immunotherapy outcomes in patients with cancer. Certain chemotherapeutic agents can extensively modify the tumor microenvironment (TME), resulting in the reprogramming of local immune responses. Although chemotherapeutic agents with an enhanced generation of potent anti-tumor immune responses have been tested in preclinical animal models and clinical trials, this strategy has not yet shown substantial therapeutic efficacy in selected difficult-to-treat cancer types. In addition, the efficacy of chemotherapeutic agent-based monotherapy in eliciting a long-term anti-tumor immune response is restricted by the immunosuppressive TME. To enhance the immunomodulatory effect of chemotherapy, researchers have made many attempts, mainly focusing on improving the targeted distribution of chemotherapeutic agents and designing combination therapies. Here, we focused on the mechanisms of the anti-tumor immune response to chemotherapeutic agents and enumerated the attempts to advance the use of chemo-immunotherapy. Furthermore, we have listed the important considerations in designing combinations of these drugs to maximize efficacy and improve treatment response rates in patients with cancer.

## Introduction

After years of intensive research to utilize the power of cytotoxic responses to fight cancer, we are witnessing a revolution in cancer therapy that harnesses the tumor recognition and destruction capabilities of the immune system (Principe et al., 2022). Conventional chemotherapy works by blocking the cell cycle and inducing apoptosis, which led to the development of multiple cytotoxic agents with non-specific targets, such as the synthesis of nucleic acids or proteins (Zitvogel et al., 2008). The effects of chemotherapeutic agents on the immune system have been neglected because of the use of cell culture and immune-deficient animal models (Galluzzi et al., 2020a). Recently, an abundance of preclinical literature has demonstrated that the immunomodulatory efficacy of conventional chemotherapeutic agents, including platinum-based drugs, anthracyclines, mitoxantrone, and taxanes, was much higher in immunocompetent mouse models than in their immunodeficient counterparts (Zitvogel et al., 2016). Further studies found that the activation of the immune system by chemotherapeutic agents leads to a two-pronged tumor eradication process: first, chemotherapeutic agents rely on cytotoxicity to directly destroy tumor cells (Casares et al., 2005); and second, the anti-tumor immune response produced by effector lymphocytes, such as cytotoxic T lymphocytes (CTLs), by secreting cytotoxic molecules and expressing ligands (Fas/TRAIL) that can bind to cell death receptors (Minute et al., 2020). This provides a partial understanding of the use of chemotherapeutic agents to potentiate therapeutic responses to immunotherapy and reprogram the tumor immune microenvironment.

The rise of immunotherapy has shifted public attention to a new field of immunomodulatory anti-tumor therapy. The improved clinical benefits brought by chemo-immunotherapy have further changed the long-held belief that chemotherapeutic agents are immunosuppressive (Galluzzi et al., 2020b). Hence, it is vital to identify the biological mechanisms underlying chemotherapy-induced immune stimulation and the key factors that can improve the efficacy of immunotherapy. There is increasing evidence that both dose and treatment interval are indispensable variables for the effective immunomodulatory effects of chemotherapeutic agents (Wu and Waxman, 2018; Fares et al., 2020; Lai et al., 2021). However, further preclinical research on chemotherapeutic agents at appropriate doses and treatment intervals has resulted in moderate anti-tumor immune responses and unsatisfactory results. Most drugs have difficulty producing durable immune efficacy because of their poor stability and off-target distribution *in vivo* and because of tumor heterogeneity and the persistently immunosuppressive microenvironment. To overcome these restrictive factors and improve overall anti-tumor efficacy, combinatorial regimens of chemotherapeutic agents, including immunotherapy agents, have been tested in clinical trials (Pfirschke et al., 2016). The intersection between chemotherapy and immunotherapy has been under evaluation for a long time, and several FDA-approved chemo-immunotherapy regimens have demonstrated significant advantages in real-world clinical applications. Therapies to obtain selective chemotherapy mediated by nanoformulations (e.g., nab-paclitaxel) or antibody-drug conjugates (ADCs; e.g., TDM-1, brentuximab vedotin) have been recently designed to achieve targeted distribution and local accumulation of anti-tumor drugs, showing significant alterations in the immunogenicity of the tumor microenvironment (TME) (Goldberg, 2015; Gu and Mooney, 2016; Coats et al., 2019).

In this review, we first summarized the immunomodulatory effects of chemotherapeutic agents, with a particular emphasis on immunogenicity and immuno-adjuvant effects. Additionally, we described the main factors affecting the immunomodulatory effects of chemotherapeutic agents and how the drugs can be used in new combinations to maximize treatment outcomes and improve response rates. Finally, we outlined some key considerations in the design of combinatorial regimens of chemotherapeutic agents to increase the application of chemo-immunotherapy in the future.

### Multiple immunoregulatory mechanisms of chemotherapeutic agents

**FIGURE 1:**
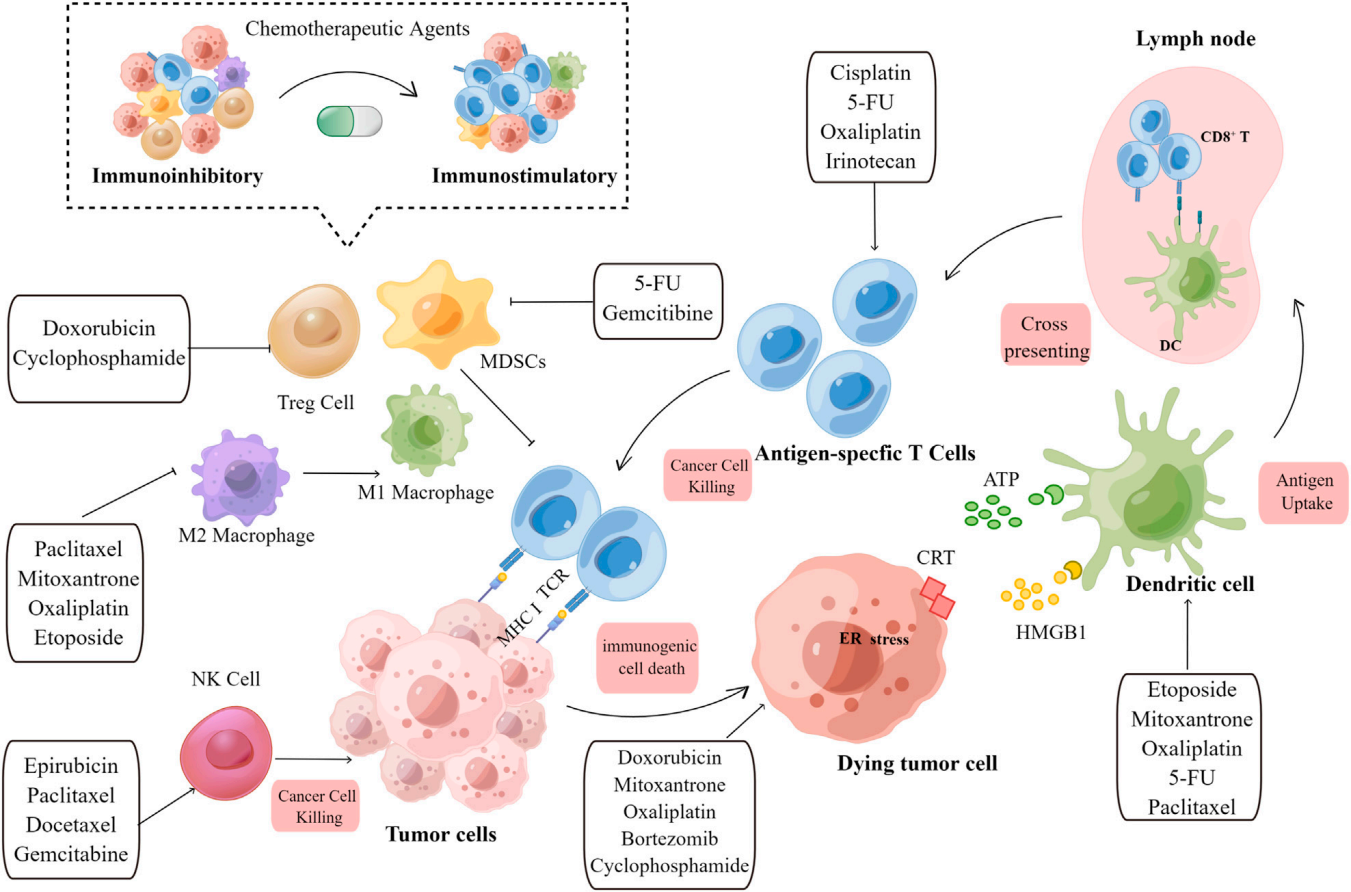
Overview of the immunostimulatory properties of chemotherapeutic agents. Immunogenic effects: When tumor cells are exposed to chemotherapeutic agents, TAA, TSA, and DAMPs released by dying tumor cells are engulfed by immature DCs, which promote APC maturation. Archived antigen-bearing APCs then migrate to the tumor-draining lymph node, where they cross-prime to T cells. Subsequently, antigen-specific T cells undergo clonal expansion. Activated T cells then recognize tumor cells and mediate the cytotoxic killing of tumor cells. Immuno-adjuvant effects: Chemotherapeutic agents can activate immune effector cells, including natural killer (NK) cells and dendritic cells (DCs), and promote the infiltration of cytotoxic T cells. The immunoinhibitory microenvironment is then transformed into an immunostimulatory microenvironment upon the depletion of immunosuppressive cells, including Treg cells, M2 macrophages, and MDSCs. Chemotherapeutic agents and their corresponding immunomodulatory effects are shown in the text boxes with arrows. TAA, tumor-associated antigen; TSA, tumor specific antigen; DAMP, damage-associated molecular pattern; DC, dendritic cell; APC, antigen-presenting cell; NK, natural killer cell; MDSC, myeloid-derived suppressor cells; Treg, regulatory T cell; MHC, major histocompatibility complex; TCR, T cell receptor; CRT,calreticulin; HMGB1, high-mobility group box 1.

The ability of chemotherapeutic agents to drive adaptive immunity depends on three main parameters: immunogenicity, adjuvanticity, and microenvironmental conditions, all of which dramatically influence neoplastic cells to develop potentially immunogenic mutations or immune susceptibility, ultimately blocking both the priming and effector phases of the immunological response (Figure 1).

## Immunogenic regulation of chemotherapeutic agents

### Eliciting immunogenic cell death

Various cytotoxic chemotherapy agents, such as anthracyclines, platinum-based drugs, mitoxantrone, oxaliplatin, and taxanes, that block cell cycle progression and induce apoptosis have been demonstrated to potentiate immunogenic cell death (ICD) (Casares et al., 2005; Kopecka et al., 2018; Li C et al., 2020; Tesniere et al., 2010; Fucikova et al., 2022). As a form of regulated cell death, ICD is amenable to activating the cellular stress response that promotes the spatiotemporally coordinated production and localization of damage-associated molecular patterns (DAMPs) from dying cells in immunocompetent hosts (Kroemer et al., 2022). DAMPs can be recognized by inherent pattern recognition receptors, such as Toll-like receptors and Nod-like receptors, on dendritic cells (DCs), which attract these cells to the tumor cells and ultimately promote tumor-associated antigen (TAA) presentation. Subsequently, antigen-presenting cells (APCs) represented by DCs, could migrate to the draining lymph nodes and present antigens to CTLs, killing tumor cells presenting the major histocompatibility complex (MHC). This event then triggers immune memory in a favorable environment with high levels of co-stimulatory signals and cytokines.

Howener, the release of DAMPs from dying cancer cells occurs during a failed intracellular stress response, and the kinetics and intensity of DAMP release can be determined through the cellular response driven by the initiating stressor (Clement et al., 2021). An increasing amount of research has confirmed that cells succumbing to intracellular stress can initiate an adaptive immune response associated with immunological memory. In contrast to chemotherapeutic agents that do not elicit ICD, most ICD inducers efficiently stimulate integrated stress responses (ISRs) (Costa-Mattioli and Walter, 2020). As a result, defects in various mechanisms associated with maintaining cellular homeostasis could influence the strength of the immune response.

ISR, a multi-pronged molecular mechanism for maintaining cellular homeostasis, is an adaptive signaling pathway activated by various forms of cellular stress, such as endoplasmic reticulum (ER) stress and the unfolded protein response (UPR) (Hetz et al., 2020; Kohli et al., 2021). One of the core molecules modulating ISR is eukaryotic translation initiation factor 2 alpha (eIF2α), whose phosphorylation is regulated by eIF2α kinase (EIF2AK1-4) (Bezu et al., 2018). Cisplatin was previously defined as incapable of triggering ICD because it does not promote the kinase-dependent phosphorylation of eIF2α or exposure of ER chaperones on the cell surface (Tesniere et al., 2010). This can be corrected by administering drugs, such as digoxin, tetracycline, that stimulate the ER stress response (Michaud et al., 2014; Xiang et al., 2020). In addition, non-phosphorylated eIF2α (eIF2αS51A) has been shown to inhibit the activation of anthracycline-driven autophagy, further demonstrating the central role of ISR in chemotherapy-induced ICD (Martins et al., 2011). This implies that one of the mechanisms underlying chemotherapeutic agent-induced ICD is ISR associated with cellular stress.

### Enhancing the antigenicity of cancer cells

While there is ample evidence that chemotherapy increases the immunogenicity of cancer cells via ICD, little is known about the chemotherapy-induced enhancement of antigenicity. In contrast to ICD, the phenotypic changes in tumor cells upon exposure to non-lethal/sublethal doses of chemotherapeutic agents are mainly manifested into enhanced antigenicity, making tumors more sensitive to CTL-mediated killing (Hodge et al., 2013). The chemotherapy-induced enhancement of tumor antigenicity is mainly associated with the upregulation of major histocompatibility complex (MHC) expression and the emergence of tumor neoantigens or TAA. Many commonly used cytotoxic agents, such as topotecan, mitoxantrone, gemcitabine, and cisplatin, have been found to upregulate the expression of antigen-presenting machinery (Grabosch et al., 2019; Li et al., 2021; Zhou et al., 2021). In addition, some chemotherapy drugs, such as docetaxel, cisplatin and 5-fluorouracil, can also promote the expression of TAA and tumor-specific antigen (TSA) and enhance antigenicity (Coral et al., 2002; Correale et al., 2003; Spehner et al., 2020). Furthermore, immune responses of CD4^+^ T cells against tumor-associated antigens were observed following oxaliplatin treatment in chemotherapy-naïve patients with mCRC (colorectal cancer) (Galaine et al., 2019). Therefore, anti-tumor T-cell responses stand out as key elements for the long-term efficacy of chemotherapy upon treatment discontinuation.

## Immuno-adjuvant effects of chemotherapeutic agents

The immune microenvironment in which cancer cells reside is a major determinant of their ability to trigger adaptive immune responses, even in the presence of sufficient antigenicity and immunogenicity (Belli et al., 2018). The immuno-adjuvant effects of the chemotherapeutic agents discussed in this review may principally rely on the recruitment and activation of immunologic effector cells to reverse the effect of an immunosuppressive microenvironment.

### Promoting dendritic cell-mediated antigen presentation

DCs have become the core initiator and regulator cells associated with anti-cancer immunity, owing to their highly complex antigen presentation mechanism. However, the abnormal evolution of tumors interferes with the mechanism of DC maturation and antigen processing in tumors, resulting in immunosuppressive effects (Murphy and Murphy, 2022). An increasing amount of evidence supports the role of chemotherapeutic agents in modulating DCs at low doses, particularly in promoting their maturation (Pfannenstiel et al., 2010; Wanderley et al., 2018) and antigen presentation capability (Shurin et al., 2009; Zhang et al., 2021). Antigen cross-presentation is a prerequisite for the induction of an effective anti-tumor immune response, and its immunomodulatory potency can be improved by apoptosis-inducing chemotherapy. McDonnell AM et al. demonstrates that gemcitabine-induced tumor cell apoptosis can increase the incidence of nuclear antigen cross-presentation *in vivo*, which is associated with an increased proportion of CTLs (McDonnell et al., 2015a; McDonnell et al., 2015b). Other drugs such as cyclophosphamide (CTX) act by altering DC biology, especially by changing DC subsets (Nakahara et al., 2010; Fumet et al., 2020).

### Trafficking and infiltration of immune effector cells

In addition, one potentially important biological response to chemotherapy is the ability to initiate T cell influx into the TME. Activated immune cells could enter the bloodstream and initiate T cell influx into the TME. *In vivo* treatment of tumor-bearing mice showed that doxorubicin, paclitaxel significantly increased the number of CD4^+^ and CD8^+^ T cells, which may be related to the expression of IFN-γ and granase B (Tsuda et al., 2007; Alizadeh et al., 2014; Heeren et al., 2019). Recent studies have shown that cisplatin can promote the production of the chemokine CCL20 and the pro-inflammatory cytokine IL-1β in tumor sites, leading to the synthesis and activation of ILC3 (Bruchard et al., 2022). ILC3 promotes the production of chemokine CXCL10 in tumors, which is associated with the generation of CD4^+^ and CD8^+^ T lymphocytes (Goc et al., 2021). It has been observed that partial chemotherapeutic agents activate NK cell-mediated anti-tumor immune responses, depending on the release of cytokines (Markasz et al., 2007; Garofalo et al., 2021). Chemotherapeutic agents also affect the composition of tumor-infiltrating lymphocytes (TILs), and it has been proven that the composition ratio between TILs and infiltrating cells is related to the efficacy of immunotherapy (Farhood et al., 2019; Saleh and Elkord, 2019). In metastatic colon cancer, multi-drug chemotherapy regimens, including FOLFOX (5-FU, folinic acid, and oxaliplatin) and FOLFIRI (5-FU, folinic acid, and irinotecan), significantly altered the composition ratio of peripheral blood lymphocytes (Maeda et al., 2011; Che et al., 2021). This change is mainly manifested by an increase in the CD8/Foxp3 TIL ratio, which can effectively predict and improve the recurrence-free and overall survival of patients. These data suggest that the immune adjuvant effect of chemotherapeutic agents runs through all stages of the cancer immune cycle and plays a role in continuous anti-tumor immune regulation.

### Depletion of immunosuppressive cells

Immunosuppressive cells, represented by myeloid-derived suppressor cells (MDSCs), regulatory T cells (Tregs), and tumor-associated macrophages (TAMs), are key influencing factors of immune escape and immunotherapy resistance and play a major role in the anti-tumor immune response (Tie et al., 2022). Interestingly, some chemotherapeutic agents have been shown to deplete immunosuppressive cell populations, thus remodeling the immune cell landscape to mount an efficient anti-tumor immune response.

The absolute or relative depletion of Treg cells, especially during tumor infiltration, is associated with the (re)induction of protective anti-cancer immunity, indicating a shift from silent or ineffective immune responses to open or effective ones (Ikegawa and Matsuoka, 2021; Nishikawa and Koyama, 2021). Previous studies on CTX-induced Treg cell depletion have obtained favorable results, confirming that CTX can mediate multiple mechanisms to selectively deplete Treg cells and further improve the effectiveness of immunotherapy by increasing the activation of autoreactive T cells (Laheurte et al., 2020). Cytokines such as IFN, IL-6, and CXCL10 play a role in CTX-mediated restoration of Treg homeostasis. CTX-induced Treg depletion and the expression of related cytokines have been shown to be directly or indirectly regulated by IFN regulatory factor (IRF)-1 (Buccione et al., 2018). In addition to CTX, many other chemotherapeutic agents selectively target Tregs. For example, patients with NSCLC who received four cycles of docetaxel chemotherapy presented fewer peripheral Tregs than at baseline, similar to that observed with cisplatin and vinorelbine (Roselli et al., 2013; Li et al., 2014).

Recent studies have shown that several aspects of macrophage biology are affected by chemotherapy. Some chemotherapeutic agents, such as CTX and doxorubicin, can activate macrophages by enhancing the secretion of GM-CSF to generate anti-tumor immune responses in mouse models of breast cancer or further potentiating Th1 responses that can enhance the tumoricidal effects of macrophages (Machiels et al., 2001; Li T et al., 2020). In addition to promoting macrophage activation, chemotherapeutic drugs can also induce macrophage polarization. For example, paclitaxel promoted the repolarization of TAMs from an M2-like to an M1-like phenotype (Wanderley et al., 2018). However, studies have similarly shown that a taxane-based chemotherapy regimen can enhance the recruitment of Tie2-expressing macrophages (TEMs) in breast cancer, promote tumor cell entry into circulation, and lead to metastasis (Karagiannis et al., 2017). Thus, the mechanism underlying the impact of chemotherapeutic agents on macrophage biology is still unclear but is most likely dependent on microenvironmental factors.

The same phenomenon can be observed with cytotoxic agents against MDSCs since many chemotherapeutic agents can selectively inhibit MDSC differentiation. Cisplatin has been shown to inhibit the conversion of monocyte precursors to inhibitory M-MDSCs through the regulation of the STAT3-COX-2 signaling axis, overcoming M-MDSC-mediated immunosuppression and improving the overall response rate to cancer immunotherapy (Van Wigcheren et al., 2021). Oxaliplatin is also known to selectively deplete MDSCs, especially Mo-MDSCs, by decreasing the expression of the immunosuppressive functional mediators argininase 1 (ARG1) and NADPH oxidase 2 (NOX2) (Kim and Kim, 2019). However, it appears that different drug combinations affect the immunomodulatory effects of chemotherapeutic drugs. Both 5-FU and gemcitabine are anti-metabolic chemotherapeutic agents that inhibit the proliferation of MDSCs by inhibiting thymidylate synthase and cytidine deaminase (Vincent et al., 2010). Interestingly, this effect was only maintained with oxaliplatin alone, and the blocking effect of MDSCs was discontinued when 5-FU was combined with irinotecan (Kanterman et al., 2014; Li et al., 2022). Given the complex regulatory effects of chemotherapeutic agents on MDSCs, this is an important area worthy of further research.

### Challenges with immunoregulatory chemotherapeutic agents to be as successful as monotherapies

**FIGURE 2:**
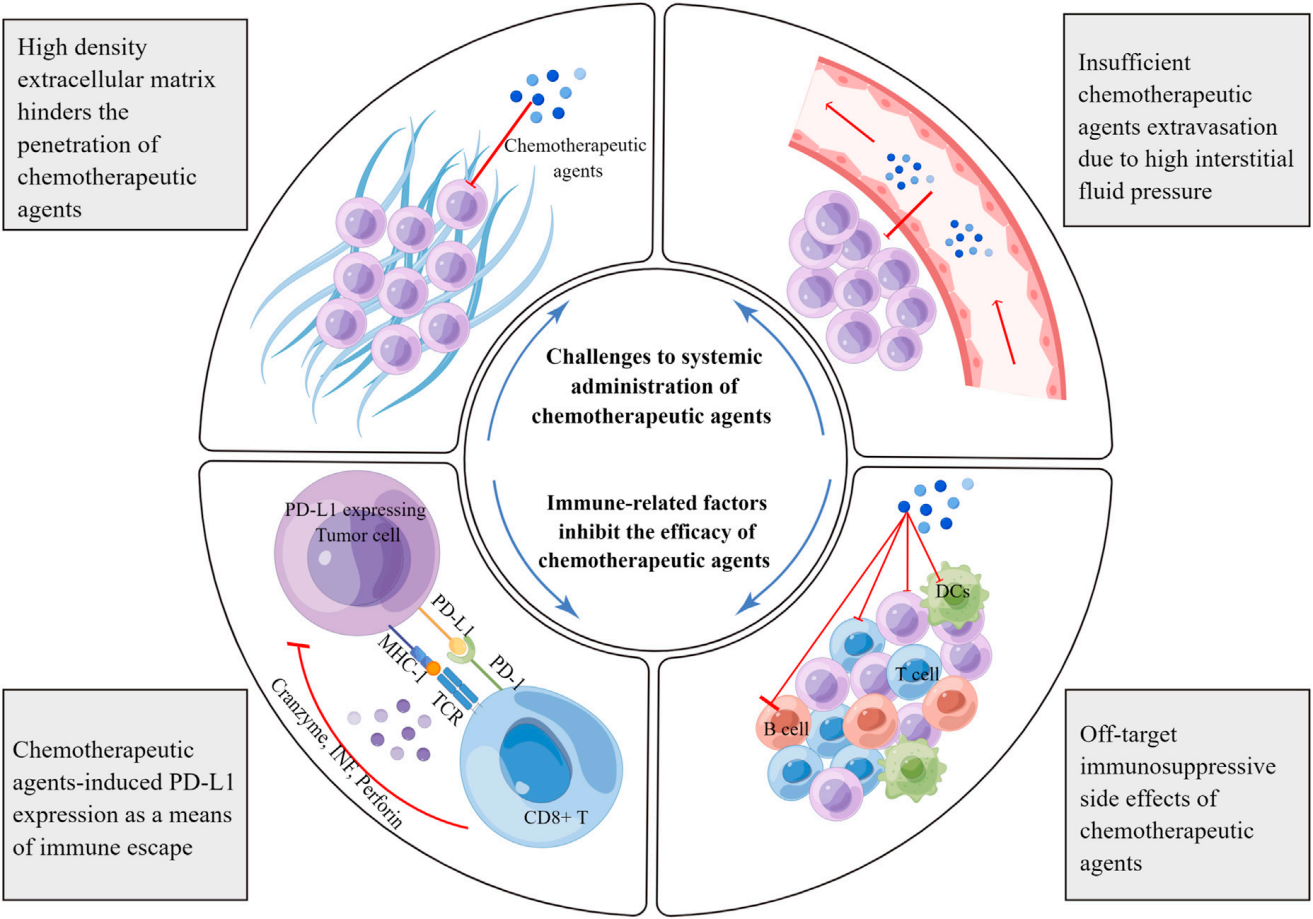
Limiting factors affecting the immunoregulatory effects of chemotherapeutic agents. Chemotherapeutic agents can induce the expression of negative immune checkpoints, especially PD-L1. PD-L1 associates with the PD-1 receptor on effector T cells, blunting anti-tumor immune responses and facilitating immune escape. Furthermore, chemotherapeutic agents target rapidly proliferating immune cells, resulting in off-target side effects. The dense network of the extracellular matrix hinders the spread of chemotherapeutic agents in tumors. In addition, there is high interstitial fluid pressure in tumor tissues, which prevents the extravasation of chemotherapeutic agents from the blood vessels. MHC, major histocompatibility complex; TCR, T cell receptor; TNF, tumor necrosis factor; DC, dendritic cell.

As mentioned above, various chemotherapeutic agents can alter the crosstalk between cancer and the immune system at multiple levels. This alteration may inhibit or kill cancer cells in an immunogenic or immuno-adjuvant-modulated manner, affect different leukocyte populations, or affect systemic physiological responses. However, it is often difficult to overcome the complexity and compensatory evolution of tumors, resulting in limited anti-tumor immune effects. Through a review of relevant studies, we have listed several key factors affecting the clinical application of chemotherapy in the following sections (Figure 2).

#### Substantial barriers that hinder the penetration of chemotherapeutic agents

Emerging evidence suggests that the TME imposes biological barriers that hinder effective cancer therapy (Binnewies et al., 2018). The structures of these biological barriers are highly irregular and are mainly characterized by a highly disordered vascular network, the absence of a lymphatic network, and high interstitial fluid pressure (Heldin et al., 2004; Jain et al., 2007). In addition, the abundant expression of extracellular matrix proteins and the proliferation of interstitial tissue in the TME constitute the mechanical barrier in cancer, resulting in insufficient tumor infiltration and obstructed blood circulation after the intravenous injection of chemotherapeutic agents (Casazza et al., 2014). Previous experiments have shown that gaps between vascular endothelial cells rarely occur in tumors in mouse models and in tumors from patients with cancer; this phenomenon is always associated with the insufficient extravasation of chemotherapeutic agents (Golombek et al., 2018). This observation suggests that trans-endothelial pathways play an integral role in the precise infiltration of chemotherapeutic agents into tumors (Sindhwani et al., 2020). To promote more efficient cancer chemotherapy in the clinic, various nanoscale drug delivery systems have been explored to achieve tumor-targeted delivery of chemotherapeutic agents. These drug delivery systems can be used to enhance the tissue-specific distribution of chemotherapeutic agents, activate immune cells, and amplify anti-tumor immune responses (Saeed et al., 2019; Wang W. et al., 2020). However, the improvement effect of traditional nanomedicine is still limited owing to the metabolic environment in tumor tissue, such as hypoxia and inflammation, which negatively affect the intratumoral immune activation effect of chemotherapeutic agents.

#### Immune resistance to chemotherapeutic agents

During tumor-host co-evolution, robust immunosuppressive circuits established in the TME hamper the ability of chemotherapeutics to drive anti-tumor immunomodulatory effects. Consequently, it is crucial to maintain the function of chemotherapeutic agents within the immunosuppressive TME. Although cisplatin, paclitaxel, and 5-fluorouracil promoted the clonal expansion of tumor-specific CD8^+^ T cells, an inadequate magnitude of an immune response was eventually manifested, owing to the increased expression of microenvironment-related IDO (Munn and Mellor, 2016). Additionally, oxaliplatin has been shown to suppress T-cell responses by promoting PD-L1 expression on DCs and reducing the expression of the co-stimulatory molecules CD80/CD86 (Tel et al., 2012). Subsequent trials have also revealed that the combination of 5-FU and oxaliplatin decreased the expression of immune checkpoints PD-L1 and PD-L2 on the surface of DCs, promoting DC maturation in tumor-bearing mice (Hong et al., 2018). Notably, the perturbation of multiple factors in the immune system drives the expression of these negative regulatory pathways, making chemotherapy monotherapy insufficient to reverse the effects of an immunosuppressive microenvironment. In addition to the influence of immunosuppressive microenvironment-related factors, the activation of unknown immune-related signaling pathways during treatment is also a key factor that inhibits the anti-tumor efficacy of chemotherapeutic drugs. Recent studies have shown that cessation of immunogenic chemotherapy may provoke the restoration of the immunosuppressive microenvironment and the immune escape of tumor cells, which could accelerate the malignant progression of tumors. Gemcitabine, a standard chemotherapy regimen for advanced pancreatic ductal carcinoma, induces the infiltration of pro-inflammatory macrophages into the liver and activates cytotoxic T cells. However, Bellomo et al. experimentally observed that after cessation of the standard chemotherapy regimen, tumor cells recruited growth- and arrest-specific 6 (Gas6)-expressing neutrophils to the liver through the secretion of CXCL1 and CXCL2, resulting in liver metastases (Bellomo et al., 2022).

#### Off-target immunosuppressive side effects of chemotherapeutics

Many chemotherapeutic agents have notable immunosuppressive side effects. These effects occur either directly by inhibiting or killing effector cells, characterized by rapid proliferation, or indirectly by inducing anergy or immune paralysis (Zitvogel et al., 2008). The off-target effects of chemotherapeutic agents on the immune system are extensive and could affect their therapeutic efficacy (Medzhitov and Janeway, 2002). This applies to CTX, which primarily impairs the proliferation of peripheral T cells and hinders their effector functions. In addition, post-transplant CTX alters immune signatures and leads to impaired T cell reconstitution in allogeneic hematopoietic stem cell transplant by increasing Treg while reducing naïve T cells (Zhao et al., 2022). Current research suggests that the number and function of immune-infiltrating effector cells are closely related to the efficacy and prognosis (Denkert et al., 2018; Paijens et al., 2021); similarly, the depletion of non-targeted immune cells affects the anti-tumor effect of chemotherapy. Clinically, owing to myelosuppression and its related immunosuppressive adverse effects, the maximum tolerated dose of chemotherapeutic agents is rarely used for the routine treatment of patients with cancer. However, it has also been suggested that the recovery phase from chemotherapy-induced lymphopenia can serve as an important window to enhance the anti-tumor immune response, providing a reasonable and feasible theoretical support for the application of chemotherapy followed by immunotherapy (Williams et al., 2007; Moschella et al., 2011). Hence, the role of transient immunosuppression in long-term immune responses and immune memory needs to be further demonstrated.

To generate a strong and durable anti-tumor immune response, it is necessary to modify the chemotherapeutic agents themselves or design a more efficient combination regimen. This can be done by changing the distribution of the chemotherapeutic drug in the body or using a combination of drugs that can better target the tumors of interest, activating the anti-tumor immune response, improving the immune response rate, and reducing the incidence of non-target immune-related side effects.

## Attempts to improve the immunomodulatory effect of chemotherapeutic agents

### Antibody-drug conjugates

ADCs were originally designed to exploit the exquisite specificity of antibodies to deliver targeted and potent chemotherapeutics. ADCs undergo a complex sequence of drug internalization procedures that often require intracellular processing and eventual payload release (Drago et al., 2021). Emerging evidence suggests that the payloads act as the workhorse of ADCs, exerting tumor-killing effects through complex interactions between ADCs and various components of the tumor immune microenvironment. A variety of cytotoxic drugs and their derivatives were used in the design of ADC drugs, which are mainly divided into the following categories: microtubule inhibitors/stability disruptors, such as auristatin derivatives (monomethyl auristatin F [MMAF] or monomethyl auristatin E [MMAE]); Mayden derivatives (DM1/DM4); and calicheamicin and its derivatives that act on DNA grooves or breaks in the double helix. Some ADC payloads, such as MMAF/MMAE, auristatins, pyrrolobenzodiazepines, and anthracycline T-PNU, have been identified as potent substances that could promote DC maturation and activate antigenic responses (Muller et al., 2014a; D'Amico et al., 2019). Additional studies have shown that cytotoxic chemotherapeutics, such as dolastatin, MMAE, and maytansinoid, are commonly used as ADC payloads to stimulate CD8^+^ effector cell migration to experimental tumors grown in mice (Muller et al., 2014b).

Several experiments have confirmed the immunomodulatory effects of the payload-based ADCs. For example, brentuximab vedotin (SGN-35) comprises an anti-CD30 antibody conjugated to vcMMAE via a protease-cleavable linker. Immunohistochemical analysis of Hodgkin’s lymphoma tumor samples from patients treated with SGN-35 revealed significant changes in the number of intra-tumoral CD8^+^ effector T cells (Theurich et al., 2013). Furthermore, when used alone or in combination with an anti-PD-L1 antibody, ADCs that target EphA2 molecules in combination with a tubulin payload could induce ICD, showing a strong ability to induce CD8^+^ T-cell infiltration (Muller et al., 2015; Agostinetto et al., 2022). These studies provide evidence that ADC-destabilizing drugs stimulate the cancer immunity cycle. Recently, the FDA-approved drug belantamab mafodotin (GSK2857916), an ADC that targets B-cell maturation antigen (BCMA) in multiple myeloma cells using MMAF as a payload, induced ICD in BCMA-expressing cancer cells and promoted DC activation *in vivo* (Yu et al., 2020). In a syngeneic mouse model, GSK2857916 was shown to induce ICD, promoting the intratumoral enrichment of TILs and T cell-dependent anti-tumor activity (Montes de Oca et al., 2021). To further optimize the targeted immunomodulatory effects of ADCs, researchers have screened potential targets of ADCs that are not just limited to malignant cells. Based on this, a CD25-targeted pyrrolobenzodiazepine dimer-based ADC was investigated for its ability to deplete Tregs and eradicate established tumors (Zammarchi et al., 2020).

In addition to ADC monotherapy, numerous ongoing or completed clinical trials have demonstrated the clinical efficacy of ADC combined with chemotherapy (Table 1), which showed good efficacy and safety in hematologic tumors and breast cancer. However, there are many challenges in the development of this type of drug. For example, the selection of potential targets, improvement of potential toxicity caused by off-target effects, and development of cytotoxic drugs with other mechanisms of action require numerous experiments to verify.

**TABLE 1:** Clinical trials on combination regimens of ADC and chemotherapy in cancers.

Drugs	Comibinations	Phase	Status	Cancer	References
Gemtuzumab Ozogamicin	Mitoxantrone + Etoposide	II	Recruiting	Acute Myeloid Leukemia	NCT03839446
	CPX-351(Liposome-encapsulated Daunorubicin-Cytarabine)	I	Recruiting	Acute Myeloid Leukemia	NCT03904251
	Tretinoin + Arsenic Trioxide	II	Recruiting	Acute Promyelocytic Leukemia	NCT01409161
	Fludarabine + high-dose cytarabine + filgrastim-sndz + idarubicin	II	Recruiting	Acute Myeloid Leukemia/High-Risk Myelodysplastic Syndrome	NCT00801489
Brentuximab vedotin	Bendamustine	II	Recruiting	Follicular Lymphoma	NCT04587687
	CHEP (Cyclophosphamide + Doxorubicin + Etoposide + Prednisone tablet)	II	Recruiting	Peripheral T-cell Lymphomas	NCT05006664
	CHP (Cyclophosphamide + Doxorubicin + Prednisone tablet)	II	Recruiting	Peripheral T-cell Lymphoma	NCT04569032
	Lenalidomide + rituximab	III	Recruiting	Diffuse Large B-cell Lymphoma	NCT04404283
	Doxorubicin + vinblastine + dacarbazine	III	Not yet recruiting	Hodgkin Lymphoma	NCT04685616
	Pralatrexate + cyclophosphamide + Doxorubicin + Prednisone	I	Recruiting	NK T-cell Leukemia/Lymphoma	NCT03719105
Ado-trastuzumabemtansine (T-DM1)	Atezolizumab + paclitaxel + trastuzumab + docetaxel	II	Recruiting	Neoplasm Metastasis	NCT00781612
	Methotrexate + hydrocortisone + cytarabine	II	Recruiting	Acute Lymphoblastic Leukemia	NCT03913559
Inotuzumab ozogamicin	mBFM (Cyclophosphamide + cytarabine + mercaptopurine + leucovorin calcium + vincristine)	II	Recruiting	B-Lymphoblastic Lymphoma or Relapsed or Refractory CD22 Positive B Acute Lymphoblastic Leukemia	NCT02981628
	Etoposide + Doxorubicin + Vincristine + Prednisoneand Cyclophosphamide	I	Recruiting	B-cell Acute Lymphoblastic Leukemia	NCT03991884
Polatuzumab Vedotin	Rituximab + Ifosfamide + Carboplatin + Etoposide (PolaR-ICE)	II	Recruiting	Diffuse Large B-Cell Lymphoma	NCT04665765
Sacituzumab govitecanhziy	Pembrolizumab + Carboplatin/Cisplatin	II	Recruiting	Non-small Cell Lung Cancer	NCT05186974
	Cyclophosphamide + N-803+PD-L1 t-haNK	I/II	Recruiting	Advanced Triple Negative Breast Cancer	NCT04927884

This table is according to https://clinicaltrials.gov/

### Nanosized drug delivery systems based on the tumor microenvironment

Owing to the negative role of the TME in compromising the therapeutic response of various cancer therapies, therapeutic modalities targeting the TME appear to enhance the overall response rate of patients to cancer treatment (Tang et al., 2021). Nano-drug delivery systems, which provide a prerequisite for the TME-targeted delivery of chemotherapeutic agents, are a promising strategy to address these challenges, because of their adequate circulation time, increased intratumoral accumulation and retention, efficient uptake by tumor cells, and precise release at tumor tissues (Weber and Mule, 2015; Abdou et al., 2020). Depending on the particular metabolic characteristics of the TME, such as hypoxia, acidic pH, overexpressed enzymes (e.g., matrix metalloproteinases 2 and 9 [MMP-2 and-9], hyaluronidase, legumain, or cathepsin B), reactive oxygen species (ROS), or glutathione (GSH), different responsive nanomaterials have been developed. These nanomaterials were initially applied to load chemotherapeutic agents and achieve intratumoral local delivery. Generally, these nanoparticles remain intact in the blood circulation to protect their payloads from degradation or leakage until they are activated by the TME (Zhou et al., 2020). The loaded chemotherapeutic agents are subsequently released into local tumor tissues, focusing on the modulation of immune-related molecules that normalize immune responses and induce ICD to restore the ability of CTLs to eradicate tumor cells.

Wang et al. developed an ROS-responsive hydrogel loaded with gemcitabine that showed significant therapeutic efficacy against both primary tumors and distant metastases. In response to ROS signaling in the TME, the released gemcitabine reduced the percentage of immunosuppressive cells, including MDSCs and M2 macrophages, while increasing PD-L1 expression in cancer cells, T cells, macrophages, and DCs. In turn, this increased PD-L1 expression synergizes with the effect of anti-PD-L1 antibodies to improve immunotherapy effect (Wang et al., 2018). A pH-responsive nanoparticle co-loaded with the ICD inducer doxorubicin was designed to induce potent ICD in cancer cells while synergistically limiting the production of immunosuppressive kynurenine by binding to alkylated NLG919 (an inhibitor of indoleamine 2,3-dioxygenase 1). Moreover, the introduction of pH-responsive nanoparticles could enable deep intratumoral penetration of therapeutic agents and effectively neutralize low pH levels, reversing the immunosuppressive effect of the TME (Zhu et al., 2020). Furthermore, Koo AN et al. developed GSH-responsive core-shell nanoparticles carrying docetaxel for selective delivery in tumor-bearing mice. These self-assembled nanoscale coordination polymer core-shell nanoparticles are cleaved by GSH in tumor cells, releasing docetaxel, and leading to T-cell priming and anti-tumor effects (Koo et al., 2012). However, complex nanosystems face hinderance such as possible interactions between different components and overall stability, which may also limit the immunomodulatory effects of chemotherapeutic drugs. Therefore, it is necessary to determine the best combination scheme to ensure that the targeted effects of chemotherapy drugs are maintained.

### Chemotherapy combined with PD-L1/PD-1 inhibitors

Since the early 2010s, many immunotherapies based on immune checkpoint inhibition have been developed, especially antibodies targeting the PD-1/PD-L1 pathway, thereby changing the status quo of cancer treatment. Tumors respond poorly to immunotherapy despite their revolutionary efficacy, which can be explained by exhibiting a loss of lymphocyte infiltration (NK, CD8, Th1), the so-called “cold tumors.“(Fumet et al., 2020) As an immune adjuvant, chemotherapy can increase the number of TILs, reverse the cold immune environment, and reduce the enrichment of immunosuppressive cells, significantly improving the response rate to immune checkpoint inhibitors (Park et al., 2020). Moreover, several chemotherapeutic agents, such as cisplatin, oxaliplatin, doxorubicin, and paclitaxel, can also enhance the expression of immune checkpoints, mostly PD-L1 (Ng et al., 2018). In this context, the expression of PD-L1 on cancer cells can associate with the PD-1 receptor on effector T cells, reducing anti-tumor immune responses and facilitating immune escape (Li et al., 2021).

Harnessing the broad immunostimulatory capabilities of chemotherapeutic agents in combination with immune checkpoint inhibitors has shown great promise with improved clinical outcomes. The FDA has already approved combinations of chemotherapy and PD-L1/PD-1 therapy (Gandhi et al., 2018; Horn et al., 2018; Schmid et al., 2018). Numerous phase III trials are currently underway for various cancer types that will alter the face of oncology for multiple indications. Our review has focused on the administration sequence and dosing schedule of combined immunochemotherapy.

Immunochemotherapy combinations currently comprise adjuvant standard-of-care chemotherapy regimens being added to immunotherapy, but the elements in this combination have not been optimized. With the deepening of the clinical application of chemotherapy combined with Immune checkpoint inhibitors, more studies began to focus on practical clinical issues such as the schedule, interval, and cycle of the combination therapy to obtain better clinical benefits (Principe et al., 2022; Leonetti et al., 2019). Studies have shown that neoadjuvant chemotherapy plays an important role in cancer treatment, and a large number of clinical trials are currently underway to evaluate the mechanism of action of immunochemotherapy regimens in neoadjuvant therapy (Table 2). In addition, increasing attention has been given to the sequence of administering anti-PD-1 antibodies and chemotherapeutic agents. A recent phase II study demonstrated that chemotherapy prior to anti-PD-L1 treatment could exert a better immunomodulatory effect in the neoadjuvant treatment of locally advanced esophageal squamous cell carcinoma (Fukuoka et al., 2019). Although this study is still ongoing, the current results suggest that delaying toripalimab administration to day 3 in immuno-chemotherapy may achieve higher pCR rates than administering both agents on the same day. This conclusion requires further large-sample clinical trials for verification.

**TABLE 2:** Clinical trials on neoadjuvant immuno-chemotherapy in cancer.

Drugs	Neoadjuvant chemotherapy	Phase	Status	Cancer	References
Pembrolizumab	Paclitaxel + Cisplatin	II	Not yet recruiting	Esophageal Squamous Cell Carcinoma	NCT05281003
	Cisplatin+5-FU	II	Recruiting	EGJ Adenocarcinoma	NCT04813523
	Gemcitabine + Cisplatin	III	Recruiting	Cisplatin-eligible Muscle-invasive Bladder Cancer (MIBC)	NCT03924856
	Folfirinox (Oxaliplatin + Leucovorin + Irinotecan+5-Fluorouracil)	II	Not yet recruiting	Pancreatic Ductal Adenocarcinoma	NCT05132504
	Nab-paclitaxel + doxorubicin + Cyclophosphamide + Carboplatin + Paclitaxel	I	Completed	Triple Negative Breast Cancer	NCT02622074
	Decitabine + dose-dense AC + paclitaxel (or paclitaxel plus carboplatin)	II	Recruiting	Locally Advanced HER2- Breast Cancer	NCT02957968
	mFOLFOX6	II	Recruiting	Gastroesophageal Junction (GEJ) and Stomach Adenocarcinoma	NCT03488667
Atezolizumab	paclitaxel + carboplatin	I/II	Completed	Advanced-Stage Ovarian Cancer	NCT03394885
	cisplatin + gemcitabine	II	Not yet recruiting	Bladder Cancer	NCT04630730
	Carboplatin + Etoposide	II	Not yet recruiting	Limited-Stage Small Cell Lung Cancer	NCT04696939
	cisplatin/carboplatin + pemetrexed (for non-squamous only)				
	cisplatin/carboplatin + gemcitabine (for squamous only)				
	carboplatin + paclitaxel	II	Recruiting	Previously Untreated Locally Advanced Resectable Stage II, IIIA, or Select IIIB Non-Small Cell Lung Cancer	NCT04832854
Nivolumab	paclitaxel + carboplatin ± cabiralizumab	I/II	Recruiting	Triple Negative Breast Cancer	NCT04331067
	cisplatin/carboplatin + pemetrexed (for non-squamous only)				
	cisplatin/carboplatin + gemcitabine (for squamous only)				
	carboplatin + paclitaxel	III	Recruiting	Surgically Removable Early Stage Non-small Cell Lung Cancer	NCT04025879
	paclitaxel + carboplatin	II	Recruiting	Non Small Cell Lung Cancer	NCT03838159
	Paclitaxel + Doxorubicin + Cyclophosphamide + Paclitaxel/Docetaxel	II	Completed	Inflammatory Breast Cancer (IBC)	NCT03742986
	pemetrexed + cisplatin/carboplatin	I	Recruiting	Mesothelioma	NCT04162015
Durvalumab	Paclitaxel + Carboplatin	II	Recruiting	Ovarian Cancer	NCT03899610
	Gemcitabine + Cisplatin				
	Gemcitabine + Carboplatin	II	Recruiting	Urothelial Carcinoma	
				Cancer	NCT04617756
	paclitaxel + epirubicin + cyclophosphamide	I/II	Recruiting	Luminal B HER2(-) or Triple Negative Breast Cancers	NCT03356860
	Gemcitabine + Cisplatin	II	Recruiting	Biliary Tract Neoplasms	
				Gallbladder Cancer	
				Cholangiocarcinoma	NCT04308174
	Carboplatin + nab-paclitaxel + anlotinib	II	Recruiting	Stage III Non-Small-Cell Lung Cancer	NCT04762030
	Docetaxel + oxaliplatin + S-1	II	Recruiting	Gastric or Gastroesophageal Junction Adenocarcinoma	NCT04221555
	FLOT (flurouroacil + leucovorin + oxaliplatin + docetaxel)	III	Recruiting	Gastric and Gastroesophageal Junction Cancer	NCT04592913
	carboplatin + paclitaxel				
	cisplatin + gemcitabine				
	pemetrexed + cisplatin				
	pemetrexed + carboplatin	III	Recruiting	Non-small Cell Lung Cance	NCT03800134

This table is according to https://clinicaltrials.gov/

With these general considerations related to drug scheduling, researchers should further investigate the different doses and lengths of drug-free periods required for various drugs and cancer types to improve the efficacy of immunogenic chemotherapy. This would further standardize the combination dynamics of different chemotherapy and immunotherapy drugs for different cancer types.

### Chemotherapy combined with oncolytic virus therapy

Oncolytic viruses (OVs) comprise a novel immune anti-tumor therapy that can target tumor cells and replicate within them, thereby killing tumor cells (Martin and Bell, 2018). OVs, including adenovirus, parvovirus, reovirus, coxsackie virus, and HSV, can promote the expression of DAMPs to induce ICD. The synergistic combination of OVs and chemotherapeutic agents can compensate for the current situation wherein the inability of chemotherapeutic agents to induce ICD is the limiting factor. This combination has a significantly improved efficacy in enhancing the stimulation of an anti-tumor immune response compared with single-drug treatment (Habiba et al., 2020). For example, the combination of ONYX-015 and cisplatin significantly improved OS in a tumor xenograft model, showing superior efficacy to cisplatin monotherapy (Khuri et al., 2000). In addition, combination therapy with the oncolytic HSV-1 and mitoxantrone increased the accumulation of antigen-specific CD8^+^ T cells in the tumor (Workenhe et al., 2013). Several clinical trials employing OVs in association with chemotherapeutic agents in various tumor types have been performed or are ongoing (Table 3). In a phase I/II trial of intravenous carboplatin/paclitaxel plus reovirus showed good objective responses and inhibition of tumor progression in cancer of the head and neck (Karapanagiotou et al., 2012). ONCOS-102 (previously known as CGTG-102) is an adenovirus equipped with GM-CSF. In a phase I trial, the safety and recommended dose of the oncolytic adenovirus ONCOS-102 combined with low-dose oral CTX for advanced cancer was investigated (Ranki et al., 2016). In a subsequent next phase I/II clinical trial, the safety and clinical efficacy of chemotherapy combined with ONCOS-102 in the treatment of malignant pleural mesothelioma were further evaluated (Kuryk et al., 2016). Therefore, chemotherapy combined with OV seems to provide a new therapeutic strategy for targeting the immune microenvironment since combining the two can better exert anti-tumor immune regulation.

**TABLE 3:** Clinical trials on combination regimens of OVs and chemotherapy in cancer.

Drugs	Comibinations	Phase	Status	Cancer	References
TG6002	5-flucytosine	I/II	Recruiting	Glioblastoma	NCT03294486
				Brain Cancer	
LOAd703	gemcitabine/nab-paclitaxel/atezolizumab	I/II	Recruiting	Pancreatic Cancer	NCT02705196
Enadenotucirev	Capecitabine + Radiotherapy	I	Recruiting	Locally Advanced Rectal Cancer	NCT03916510
TBI-1401(HF10)	Gemcitabine + Nab-paclitaxel or TS-1	I	Active, not recruiting	Pancreatic Cancer Stage III/IV	NCT03252808
rQNestin34.5v.2	Cyclophosphamide	I	Recruiting	Brain Tumor	NCT03152318
Talimogene laherparepvec	Paclitaxel	I/II	Active, not recruiting	Breast Cancer	NCT02779855
OH2 oncolytic virus	LP002 + Cisplatin + Fluorouracil	I	Recruiting	Digestive System Neoplasms	NCT04755543
olvimulogene nanivacirepvec(Olvi-Vec)	Platinum chemotherapy: carboplatin (preferred) or cisplatin	III	Not yet recruiting	Ovarian Cancer	NCT05281471
Pelareorep	paclitaxel + avelumab	II	Recruiting	Breast Cancer Metastatic	NCT04215146
JX-594 (Pexa-Vec)	Irinotecan	I/II	Completed	Colorectal Carcinoma	NCT01394939
Reovirus Serotype 3—Dearing Strain (REOLYSIN^®^)	chemotherapy(Gemcitabine/Irinotecan/Leucovorin+5-fluorouracil)+pembrolizumab	I	Completed	Pancreatic Adenocarcinoma	NCT02620423
Reovirus Serotype 3—Dearing Strain (REOLYSIN^®^)	Carboplatin + Paclitaxel	II	Completed	Carcinoma, Non-small Cell Lung	NCT00861627
Reovirus Serotype 3—Dearing Strain (REOLYSIN^®^)	Irinotecan/Fluorouracil/Leucovorin (FOLFIRI)+bevacizumab	I	Completed	KRAS Mutant Metastatic Colorectal Cancer	NCT01274624
oncolytic measles virus encoding thyroidal sodium iodide symporter (MV-NIS)	pegylated liposomal doxorubicin/gemcitabine/topotecan/paclitaxel + bevacizumab	II	Recruiting	Ovarian Carcinoma	NCT02364713
DNX2401	Temozolomide	I	Completed	Glioblastoma Multiforme	
				Recurrent Tumor	NCT01956734
Oncolytic Reovirus (Reolysin NSC # 729,968)	Paclitaxel	II	Completed	ovarian epithelial, fallopian tube, or primary peritoneal cancer	NCT01199263
CGTG-102	low-dose metronomic cyclophosphamide	I	Completed	Malignant Solid Tumour	NCT01598129
CGTG-102	Pemetrexed/cisplatin (carboplatin)/Cyclophosphamide	I/II	Active, not recruiting	Unresectable Malignant Pleural Mesothelioma	NCT02879669

This table is according to https://clinicaltrials.gov/

### Chemotherapy combined with cellular stress inducers

The mechanisms through which chemotherapeutic agents induce ICD are complex, diverse, and mainly related to cellular stress responses, manifested through ER stress, induction of autophagy, and ATP release (Zhou et al., 2021; Radogna and Diederich, 2018). Owing to tumor heterogeneity and the influence of the physicochemical properties of chemotherapeutic drugs, the restricted adjuvanticity of chemotherapy compromises the ability of dying cells to activate anti-tumor adaptive immunity. Recently, several cellular stress inducers have been discovered and used synergistically with chemotherapeutic agents to improve the overall response rate of ICD. As a representative cardiac glycoside, digoxin can potentially induce characteristic biomarkers of ICD, such as CRT exposure, ATP secretion, and HMGB1 release (Menger et al., 2012). When combined with cisplatin, a significant percentage of tumor-free mice were revaccinated with live tumor cells in mouse models (Xiang et al., 2020). This experimental result was confirmed to depend on the activation of anti-tumor immune responses. Likewise, statins have great potential to promote calreticulin exposure on the tumor cell surface and enhance cellular signaling associated with ER stress. In addition, statins can promote the activation and recruitment of APCs and tumor-specific CD8^+^ T cells in tumor tissues and draining lymph nodes, further enhancing the sensitivity of immune effector cells to ICD-related markers (Kwon et al., 2021). This effect was shown to be significantly improved after combined treatment with cisplatin. Moreover, chemotherapy-induced autophagy is required for the trafficking of T lymphocytes and dendritic cells. Oxaliplatin (OXA) has been experimentally shown to activate immunomodulatory responses by activating autophagy-dependent ATP release (Martins et al., 2012). On this basis, Wang et al. used the autophagy inducer Thiostrepton in combination with OXA and observed the shrinkage of TC1 NSCLC and MCA205 fibrosarcoma cells in an immunoactive C57BL/6 mouse model, which was dependent on the activation of T lymphocytes (Wang Y. et al., 2020). However, further preclinical studies focusing on the complete and dynamic biological mechanisms that account for cellular stress inducers are needed to facilitate the development of additional cell stress inducers. This would enable clinicians to strategically combine chemotherapy to induce immunogenic cellular stress.

## Conclusion

As traditional anti-cancer agents, chemotherapeutic agents have exhibited significant benefits to patients with cancer. Although long considered immunosuppressive, there is mounting evidence to support the selection of chemotherapeutic agents with immunostimulatory properties as effective anti-cancer therapies. However, despite multiple mechanisms, the critical role of chemotherapeutics in determining the overall efficacy of cancer treatment remains unclear. With an improved understanding of the association of immunology and tumor biology at the molecular level, immunogenic cell death emerges as one of several crucial mechanisms by which chemotherapeutics elicit tumor-targeted immune responses. Prospective preclinical results and preliminary clinical findings suggest that the integrated stress response induced by chemotherapeutic agents may be key to their long-term immunomodulatory effects. Further research and exploration of the mechanisms underlying the immunomodulatory effects of chemotherapy are required.

Accumulating clinical data indicate the potency of chemotherapeutics in combination with other anti-cancer drugs, particularly immunotherapy. When combining chemotherapeutic agents with immunotherapies or designing suitable delivery systems for chemotherapeutic drugs, such as nano-targeted delivery and ADCs, one should consider the relative merits of the constituent drugs in terms of their targets, pharmacokinetics, and safety. In addition, we also need to consider the administration sequence, time interval, and dose of the combination therapy. Lastly, biomarkers that can identify responses to combinatorial regimens of chemotherapeutic agents remain unknown. Recently, liquid biopsy, owing to its ability to monitor the immune landscape of the TME dynamically, appears to be useful for guiding immunogenic chemotherapy and providing a real-time biomarker screening approach. Overall, combinatorial regimens of chemotherapeutic agents are promising therapeutic platforms for optimizing combinatorial cancer treatments.

## Abbreviations

ADC, antibody-drug conjugate; ADCs, Antibody-drug conjugates; APC, antigen-presenting cell; BCMA, B-cell maturation antigen; CTL, cytotoxic T lymphocyte; CTX, cyclophosphamide; DAMP, damage-associated molecular pattern; DC, dendritic cell; ER, endoplasmic reticulum; eIF2α, eukaryotic translation initiation factor 2 alpha; GSH, glutathione; ICD, immunogenic cell death; ISR, integrated stress response; MMAE, monomethyl auristatin E; MMAF, monomethyl auristatin F; MDSCs, myeloid-derived suppressor cells; MHC, major histocompatibility complex; MMP, matrix metalloproteinases; NOX2, NADPH oxidase 2; OXA, Oxaliplatin; OVs, oncolytic viruses; ROS, reactive oxygen species; TAA, tumor-associated antigen; TAMs, tumor-associated macrophages; TEMs, Tie2-expressing macrophages; Tregs, regulatory T cells; TME, tumor microenvironment; UPR, unfolded protein response.
